# Contractile effects of stimulation of D_1_-dopamine receptors in the isolated human atrium

**DOI:** 10.1007/s00210-024-03340-z

**Published:** 2024-08-05

**Authors:** U. Gergs, T. H. Pham, L. M. Rayo Abella, C. Hesse, P. Grundig, S. Dhein, B. Hofmann, J. Neumann

**Affiliations:** 1 Institute for Pharmacology and Toxicology, Medical Faculty, Martin Luther University Halle-Wittenberg, Magdeburger Straße 4, 06112 Halle (Saale), Germany; 2 Rudolf Boehm Institute of Pharmacology and Toxicology, Medical Faculty, University Leipzig, Leipzig, Germany; 3 Department of Cardiac Surgery, Mid-German Heart Center, University Hospital Halle, Halle (Saale), Germany

**Keywords:** Human dopamine 1 receptors, Human atrium, Force of contraction

## Abstract

Dopamine receptors have been claimed not to directly increase contractility in the human heart. Therefore, we performed contraction experiments in isolated electrically driven human atrial preparations (HAP). For comparison, we performed contraction experiments with left atrial preparations of transgenic mice which harbor a cardiac overexpression of human D_1_-dopamine receptors (D_1_-TG). In D_1_-TG, first we noted that dopamine (10 nM–10 µM cumulatively applied) in the presence of propranolol exerted a concentration- and time-dependent positive inotropic effect in D_1_-TG. In a similar fashion, dopamine increased force of contraction in the presence of 0.4 µM propranolol in HAP and these effects were amplified by pre-treatment with inhibitor of phosphodiesterase III (1 µM) cilostamide. Moreover, contractile effects of dopamine in the presence of propranolol 0.4 µM in HAP were antagonized by odapipam, haloperidol, or raclopride. Ten micromolars of fenoldopam in the presence of cilostamide increased force of contraction in HAP and this effect was antagonized by SCH 23390. We conclude that stimulation of human D_1_-dopamine receptors can increase force of contraction in the HAP.

**Supplementary Information:**

The online version contains supplementary material available at 10.1007/s00210-024-03340-z.

## Introduction

**Fig. 1:**
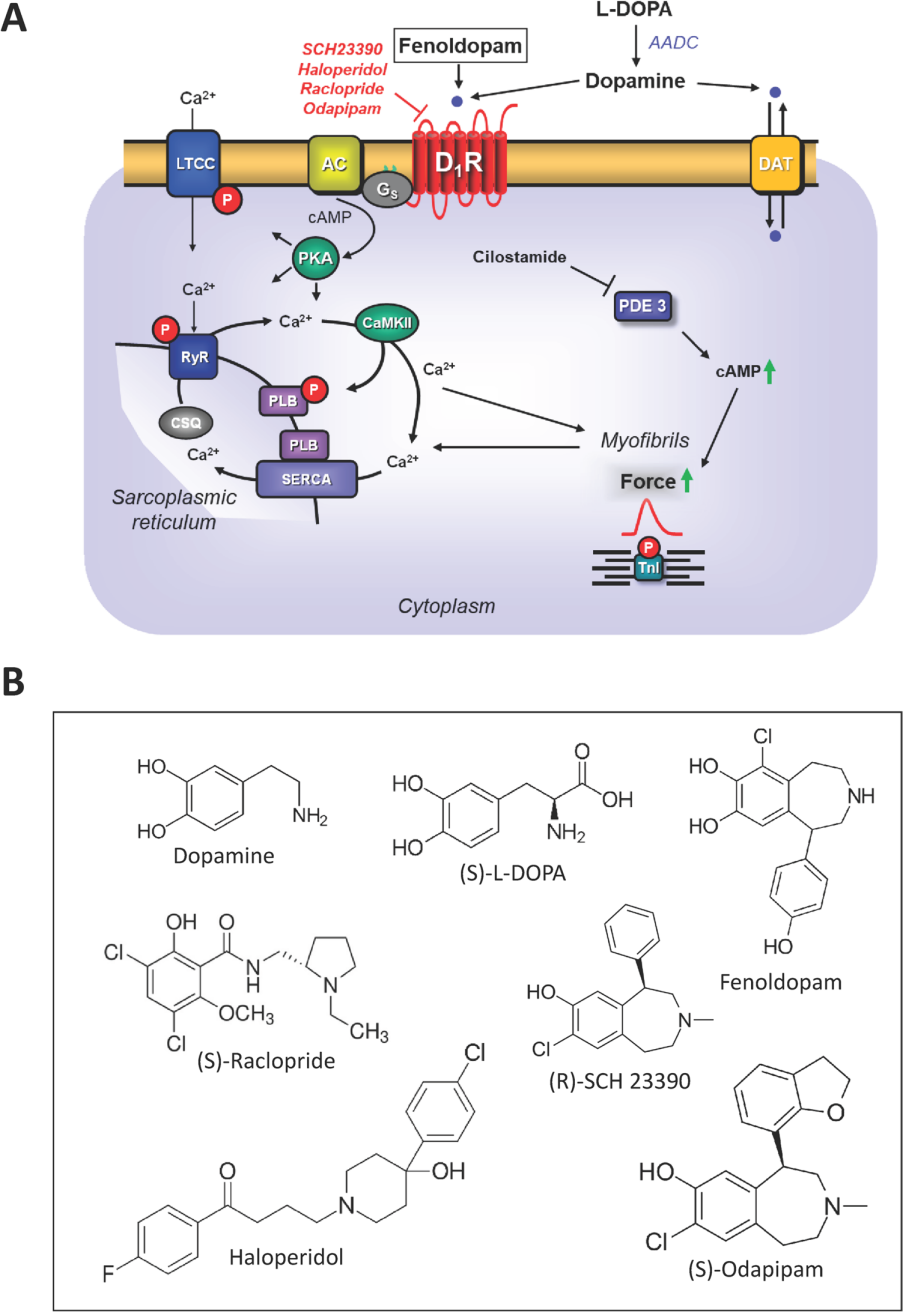
**A** Scheme of the signal transduction of D_1_-dopamine receptors in the human atrium in cardiomyocytes. Stimulation of D_1_-dopamine receptors by dopamine or fenoldopam leads to production of cAMP. The increase of cAMP leads to increases in force of contraction. The cAMP is degraded and inactivated by phosphodiesterases (PDEs). Cilostamide inhibits the main human PDE 3-isoform. The D_1_-dopamine receptors can be blocked by SCH 23390 and the listed antagonists. **B** Chemical structures of the compounds studied. The listed enantiomers for raclopride, SCH 23390 and odapipam were used from commercial suppliers

In humans, D_1_-dopamine receptors and D_5_-dopamine receptors were detected in atrium and ventricle of the heart (Amenta et al. 1993; Murphy et al. 2001; Zeng et al. 2007; Cavallotti et al. 2010; Tonnarini et al. 2011). D_1_-dopamine receptors are usually stimulated in experimental studies by dopamine or fenoldopam and are antagonized by drugs like SCH 23390, raclopride, haloperidol, or odapipam (Fig. 1A). It is impossible to differentiate with agonists or antagonists pharmacologically between D_1_-dopamine receptor-mediated effects from D_5_-dopamine receptor-mediated positive inotropic effects (review: Myslivecek 2022). For that reason, we usually speak in the remainder of this work of D_1_-dopamine receptors and mean that we do not know whether this inotropic effect is mediated by D_1_-dopamine receptors or D_5_-dopamine receptors or both.

There is very limited evidence that dopamine can directly stimulate D_1_- and/or D_5_-dopamine receptors on animal cardiomyocytes. One of the few examples is the following: in isolated rabbit ventricular cardiomyocytes, dopamine stimulated the current through the l-type calcium channel (Ding et al. 2008). This effect was D_1_-dopamine receptor-mediated based on the fact that it was stimulated by dopamine and antagonized by SCH 23390. However, contractility was not measured in these cardiomyocytes and thus a direct coupling of a dopamine receptor to force of contraction was not shown (Ding et al. 2008).

As concerns previous work on force of contraction, in HAP, dopamine is well known to increase force of contraction in vitro (Bravo et al. 1991). However, several independent labs came to the same conclusion: the contractile effects of dopamine are mediated in major part via β-adrenergic receptors in the HAP (Bravo et al. 1991; Deighton et al. 1992). In minor part, dopamine could exert positive inotropic effects in HAP via α-adrenoceptors (Kaumann et al. 1989). In addition, the positive inotropic effects of dopamine in HAP or left ventricular human muscle preparations were decreased by pretreatment of samples with cocaine (Brown et al. 1985; Deighton et al. 1992). This finding with cocaine can be explained by an indirect sympathomimetic effect of dopamine by entering cells and releasing noradrenaline and is consistent with similar mechanisms in animal tissue (Endoh et al. 1976; Brodde et al. 1980).

We were motivated to re-examine this question (Bravo et al. 1991; Deighton et al. 1992) when we succeeded in generating transgenic mice that overexpress the human D_1_-dopamine receptor (D_1_-TG, Rayo Abella et al. 2023, 2024). We could show in D_1_-TG that dopamine can increase force of contraction directly via D_1_-dopamine receptors (Rayo Abella et al. 2023, 2024). However, at that time, we could not address the question what the role of the D_1_-dopamine receptor might be in the human heart. Hence, we could not prove or rule out a positive inotropic effect of dopamine via dopamine receptors in the human heart as was questioned by the anonymous reviewer (Rayo Abella et al. 2024).

Thus, in order to ascertain a further physiological role to dopamine in the human heart, we tested in this study the hypothesis that dopamine would increase force of contraction in HAP directly via cardiac D_1_-dopamine receptors and/or D_5_-dopamine receptors. For comparison, we also studied the effect of dopamine on force of contraction in atrial preparations from D_1_-TG under identical experimental conditions. Parts of this work have been published in abstract form (Rayo Abella et al. 2023, Grundig et al. 2024).

## Materials and methods

### Contractile studies on human preparations

The contractile studies on human preparations were done using the same equipment and setup and buffer as used in the mouse studies (see below). The samples were obtained from seven male patients and two female patients with coronary heart disease (two to three vessel diseases), hypertension, and atrial fibrillation, aged 55–86 years. Drug therapy included metoprolol, furosemide, apixaban, statins, and acetyl salicylic acid. Our methods used for atrial contraction studies in human samples have been previously published and were not altered in this study (Gergs et al. 2009). Patients had given written informed consent.

### Generation of transgenic mice

Generation of D_1_-TG has been recently published (Rayo Abella et al. 2024). In brief, we overexpressed the complete cDNA of the human D_1_-dopamine receptor under the control of a heart specific promoter. Animals were crossed into a CD1 background. Mice of random sex (D_1_-TG: four females and two males; WT: one female and five males) being about 172 days of age were used. The experiments were allowed by the local animal protection institution.

### Contractile studies in mice

In brief, the right or left atrial preparations from the mice were isolated and mounted in organ baths as previously described (Neumann et al. 1998; Gergs et al. 2004). The bathing solution of the organ baths contained in millimolars the following: 119.8 NaCI, 5.4 KCI, 1.8 CaCl_2_, 1.05 MgCl_2_, 0.42 NaH_2_PO_4_, 22.6 NaHCO_3_, 0.05 Na_2_EDTA, 0.28 ascorbic acid, and 5.05 glucose. The solution was continuously gassed with 95% O_2_ and 5% CO_2_ and maintained at 37 °C and pH 7.4 (Neumann et al. 1998). Left atrial preparations were mounted vertically in 10 ml buffer containing organ baths under isometric conditions. They were stimulated with rectangular impulses with a Grass SD 9, Plain City, Ohio, USA; stimulator for a duration of 5 ms; and 10% over stimulation threshold with field stimulation using platinum electrodes. Signals were amplified via a bridge amplifier and processed using software (Labchart) from AD instruments. Spontaneously beating right atrial preparations from mice were used to study chronotropic effects. The drug application was as follows. After equilibration was reached, dopamine or fenoldopam was added to left atrial or right atrial preparations to establish concentration–response curves. Then, where indicated, antagonists (Fig. 1B) were added, where indicated propranolol was initially added to block β-adrenoceptors.

### Western blotting

The homogenization of the samples, protein measurements, electrophoresis, and primary and secondary antibody incubation and quantification were performed following our previously established protocols (Gergs et al. 2009).

The studied primary antibody against D_1_-dopamine receptors was rabbit polyclonal anti-DRD_1_ antibody from Bioss, Woburn, MA, USA (#bs-1007R, dilution 1:500).

As controls in some Western blotting experiments, cardiac homogenates from D_1_ receptor knock-out mice were used. Cardiac samples of D_1_ knock-out mice were kindly provided by Jean-Antoine Girault, Insern Research Director; Institute du Fer à Moulin, Paris, France.

### Data analysis

Data shown are means ± standard error of the mean. Statistical significance was estimated using Student’s *t*-test or the analysis of variance followed by Bonferroni’s *t*-test, as we felt appropriate. A *p*-value < 0.05 was considered to be significant.

### Drugs and materials

The drugs isoprenaline hydrochloride, dopamine hydrochloride, fenoldopam mesylate, (S)-raclopride, haloperidol, and (R)-SCH 23390 hydrochloride were purchased from Sigma-Aldrich (Dreieich Germany). (S)-Odapipam was purchased from MedChemExpress (Monmouth Junction, NJ, USA). All other chemicals were of the highest purity grade commercially available. Deionized water was used throughout the experiments. Stock solutions were prepared fresh daily.

## Results

### Contraction in human atrium

**Fig. 2:**
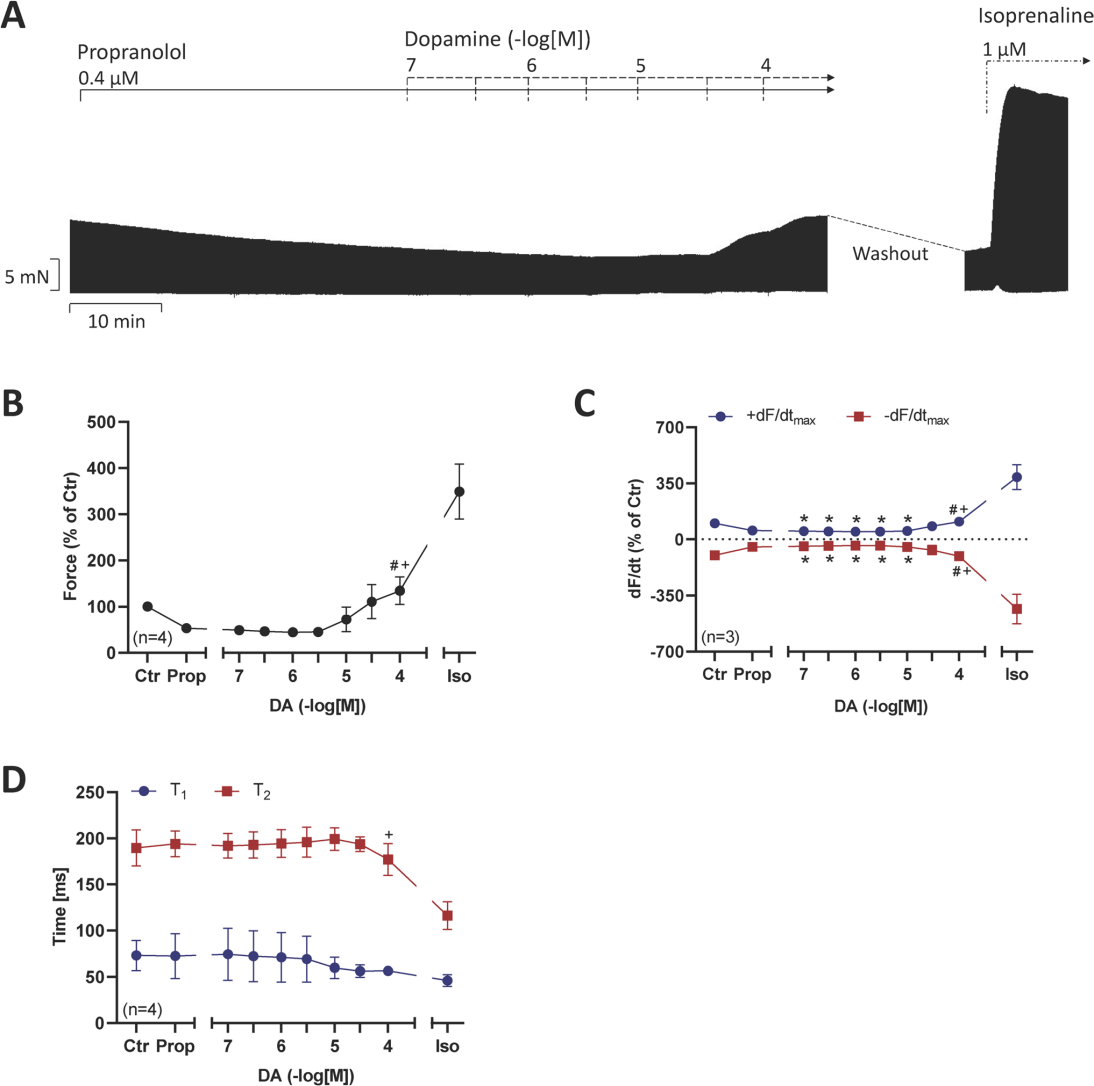
**A** Original recording of the concentration- and time-dependent positive inotropic effect of dopamine in mN (Ordinate) in electrically stimulated human right atrial muscle strips. Horizontal bar indicates time axis in min. In samples, we added 0.4 µM propranolol to the organ bath in order to block β-adrenoceptors. Note that the increase in force is slow under dopamine compared to isoprenaline (1 µM). **B** Force of contraction, **C** rate of contraction and rate of relaxation (+ dF/dt_max_ and − dF/dt_max_), **D** time to peak tension (T_1_) and time to relaxation (T_2_). Statistical significance was estimated using the analysis of variance followed by Bonferroni’s *t*-test. *p < 0.05 vs. control (Ctr), ^#^*p* < 0.05 vs. propranolol (Prop); ^+^*p* < 0.05 vs. isoprenaline (Iso). Numbers in brackets indicate number of experiments. Ordinate in panel **A**: force of contraction in mN. Ordinate in panels **B** and **C** in % of pre-drug value (control: Ctr). Ordinate in panel **D** in ms. Abscissae indicate molar concentrations of dopamine in negative decadic molar concentrations

**Fig. 3:**
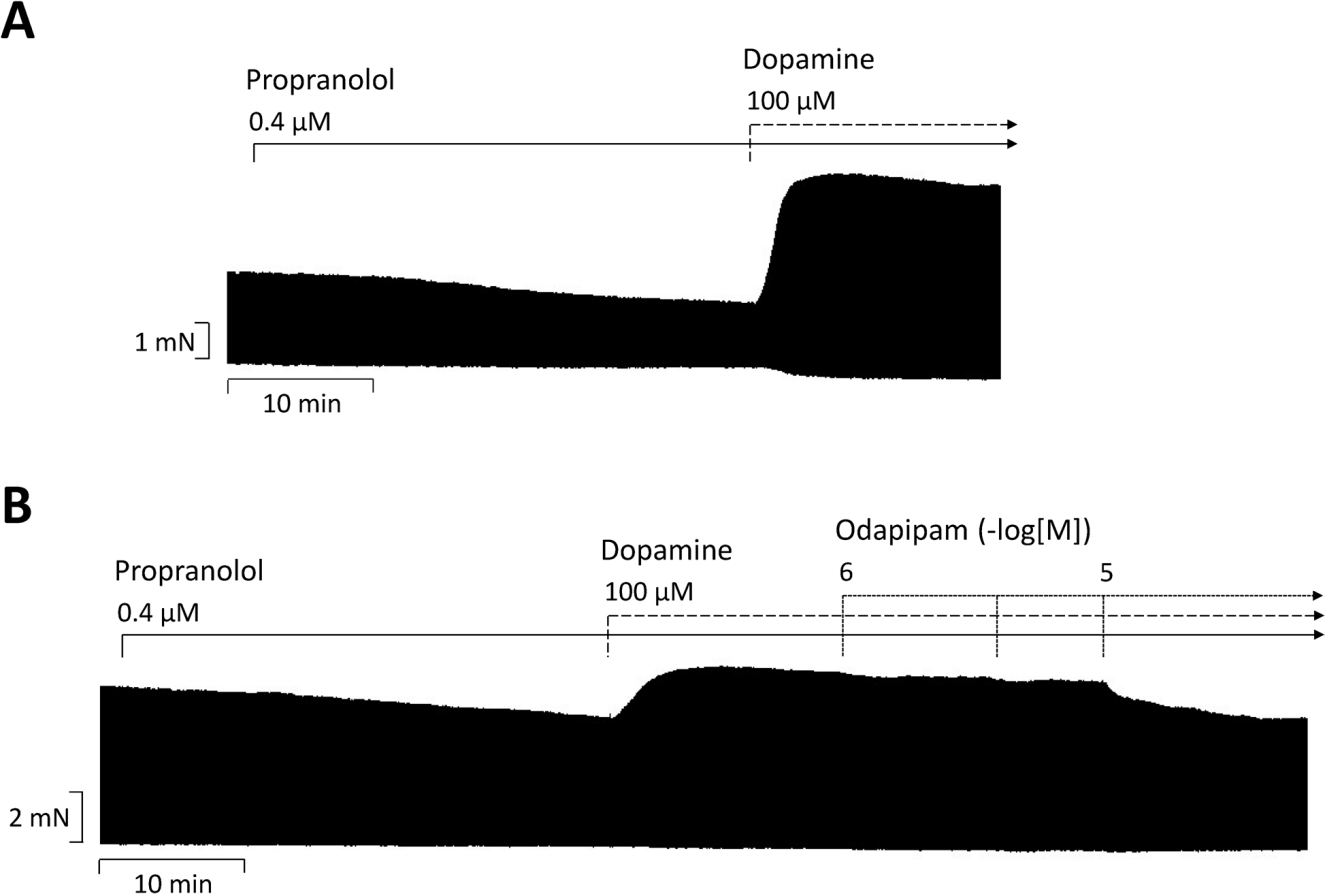
**A** Original recording of the time-dependent positive inotropic effect of dopamine in mN (Ordinate) in electrically stimulated human right atrial muscle strips. Horizontal bar indicates time axis in min. In samples, we added 0.4 µM propranolol to the organ bath in order to block β-adrenoceptors. **B** After dopamine, increasing concentrations of odapipam were added to the organ bath

**Fig. 4:**
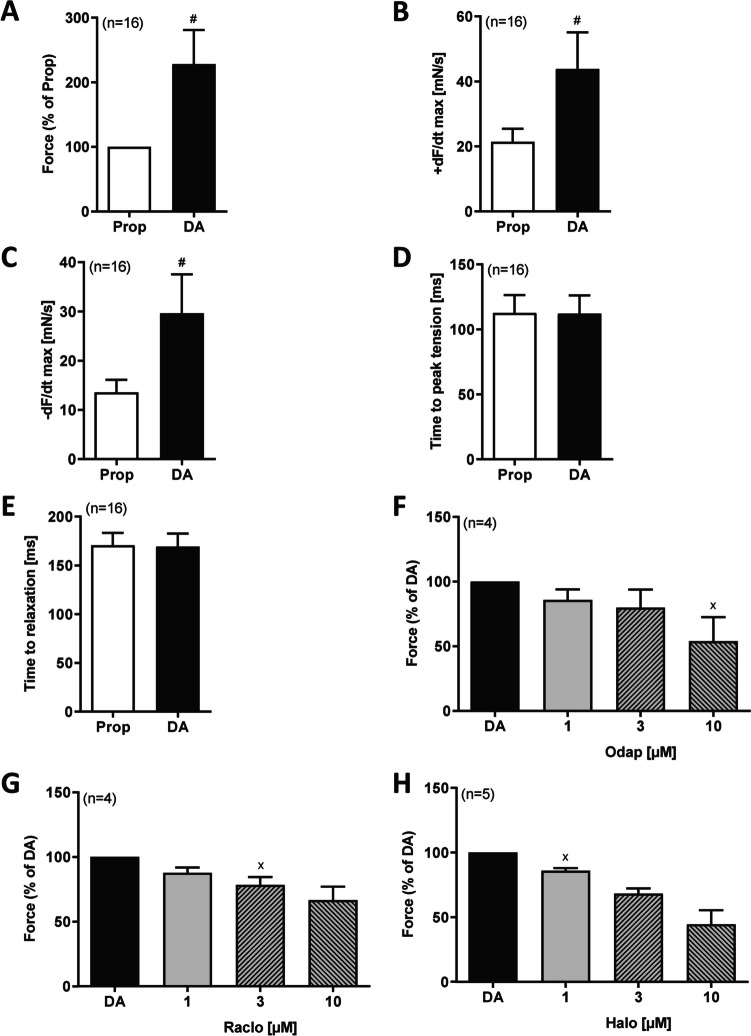
Summarized increase of force of contraction by 100 µM dopamine (DA) in % of propranolol value (Prop, **A**), **B** rate of contraction (+ dF/dt_max_), **C** rate of relaxation (− dF/dt_max_), **D** time to peak tension, **E** time to relaxation. Reduction of increase in force of contraction induced by 100 µM DA by odapipam (Odap, **F**) or raclopride (Raclo, **G**) or haloperidol (Halo, **H**). Statistical significance was estimated using Student’s *t*-test. ^x^first *p* < 0.05 vs. dopamine (DA), ^#^*p* < 0.05 vs. propranolol (Prop). Numbers in brackets indicate number of experiments. Abscissae indicate µM concentrations of drugs

As seen in the original recording in Fig. 2A, in the presence of 0.4 µM propranolol (used to block β-adrenergic effects of dopamine), high concentrations of dopamine exerted a time-dependent and concentration-dependent positive inotropic effect. This effect gained significance at 100 µM dopamine. After washout of drugs, we added 1 µM isoprenaline. This indicates that dopamine is less potent and effective to raise force of contraction in HAP than the β-adrenoceptor agonist. These effects are summarized in % of pre-drug value (Fig. 2B) or dF/dt (Fig. 2C). Time to peak tension and time of relaxation was plotted in Fig. 2D. Of note, dopamine at concentrations raised force (Fig. 2B), while isoprenaline shortened time of relaxation (Fig. 2D). As the effect of dopamine was only significant at 100 µM, this concentration alone was studied further. As seen in an original recording in Fig. 3A, dopamine raised force of contraction in the presence of 0.4 µM propranolol. This increase in force of contraction referred to propranolol (considered as pre-drug value) is given in Fig. 4A. Moreover, as in Fig. 3, also when we applied only the single concentration of 100 µM dopamine, this led to an increase in absolute values of the rate of tension development (Fig. 4B) or the rate of relaxation (Fig. 4C). Like in Fig. 3, time parameters were not shortened (Fig. 4D and E).

Next we wanted to confirm that this positive inotropic effect of 100 µM dopamine is really mediated by dopamine receptors. As there is not a single specific receptor antagonist, we tried three structurally different antagonists (Fig. 1B for their structures). A typical original experiment is given in Fig. 3B. At the start, propranolol was given to block β-adrenoceptors then 100 µM dopamine was applied to raise force of contraction and then we gave increasing concentrations of odapipam (Fig. 3B). Data from several experiments with odapipam are summarized in Fig. 4F and indicate that odapipam, a somewhat selective D_1_-dopamine receptor antagonist, antagonized the positive inotropic effect of dopamine. Similar reductions in dopamine-induced force of contraction in HAP were found for raclopride (Fig. 4G) and haloperidol, an unselective D_1_- and D_2_- dopamine receptor antagonist (Fig. 4H).

Having only used dopamine as an agonist, the question arose whether other agonists at D_1_-dopamine receptors could repeat the findings with dopamine. We chose therefore fenoldopam, because previous workers used fenoldopam as an agonist and because fenoldopam is an approved drug in humans and thus gives additional translational relevance to our study.

**Fig. 5:**
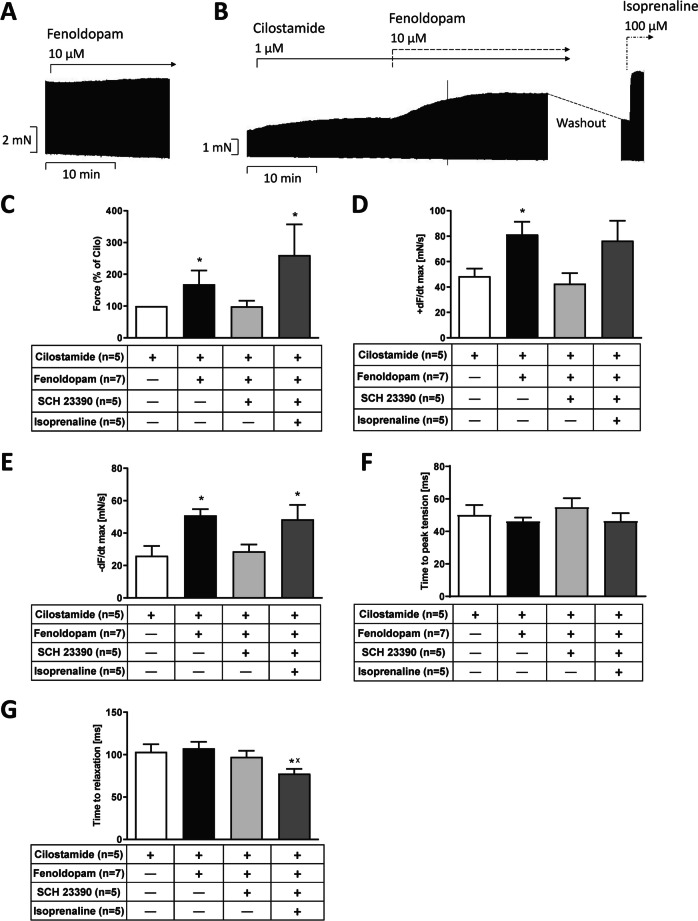
Original recording of the time-dependent positive inotropic effect of fenoldopam in mN (Ordinate) in electrically stimulated human right atrial muscle strips in the presence (**B**) or the absence (**A**) of 1 µM cilostamide and 100 µM isoprenaline. Horizontal bars indicate time axis in min. Note that the increase in force is slow under fenoldopam compared to isoprenaline (100 µM). **C** Force of contraction, in % of the effect of cilostamide (Cilo). **D** Rate of contraction (+ dF/dt_max_), **E** rate of relaxation (− dF/dt_max_), **F** time to peak tension, **G** time to relaxation. Statistical significance was estimated using Student’s *t*-test. *p < 0.05 vs. cilostamide, ^x^*p* < 0.05 vs. fenoldopam. Numbers in brackets indicate number of experiments. Ordinate in panels **A** and **B**: force of contraction in mN. Rate of contraction and rate of relaxation in panels **D** and **E** in mN/s. Ordinates in panels **F** and **G** in ms. SCH 23390 was given at the end at 1 µM

Usually, fenoldopam alone failed to increase force of contraction but in one HAP we detected a small effect of fenoldopam alone (original recording (Fig. 5A)). Only in the presence of the PDE 3 inhibitor cilostamide, fenoldopam always exerted a positive inotropic effect (original recording in Fig. 5B). This effect was weaker than that of 100 µM isoprenaline, a supramaximal concentration at the β-adrenoceptor (original recording in Fig. 5B).

These positive inotropic effects of fenoldopam are summarized in the presence of cilostamide. They are separately given in bar diagrams for force of contraction (Fig. 5C), for the rate of tension development (Fig. 5D) or the rate of relaxation (Fig. 5E). Like for dopamine (Fig. 4D and E), also fenoldopam failed to shorten time to peak tension and time of relaxation (Fig. 5F and G). The effect of fenoldopam on force of contraction (Fig. 5C) and the rate of tension development (Fig. 5D) and the rate of relaxation (Fig. 5E) were antagonized by SCH 23390. Subsequently applied isoprenaline (used as a control) raised force of contraction (Fig. 5C), the rate of tension relaxation (Fig. 5E), and shortened time of relaxation (Fig. 5G).

### Contraction in left atrium of D_1_-TG

**Fig. 6:**
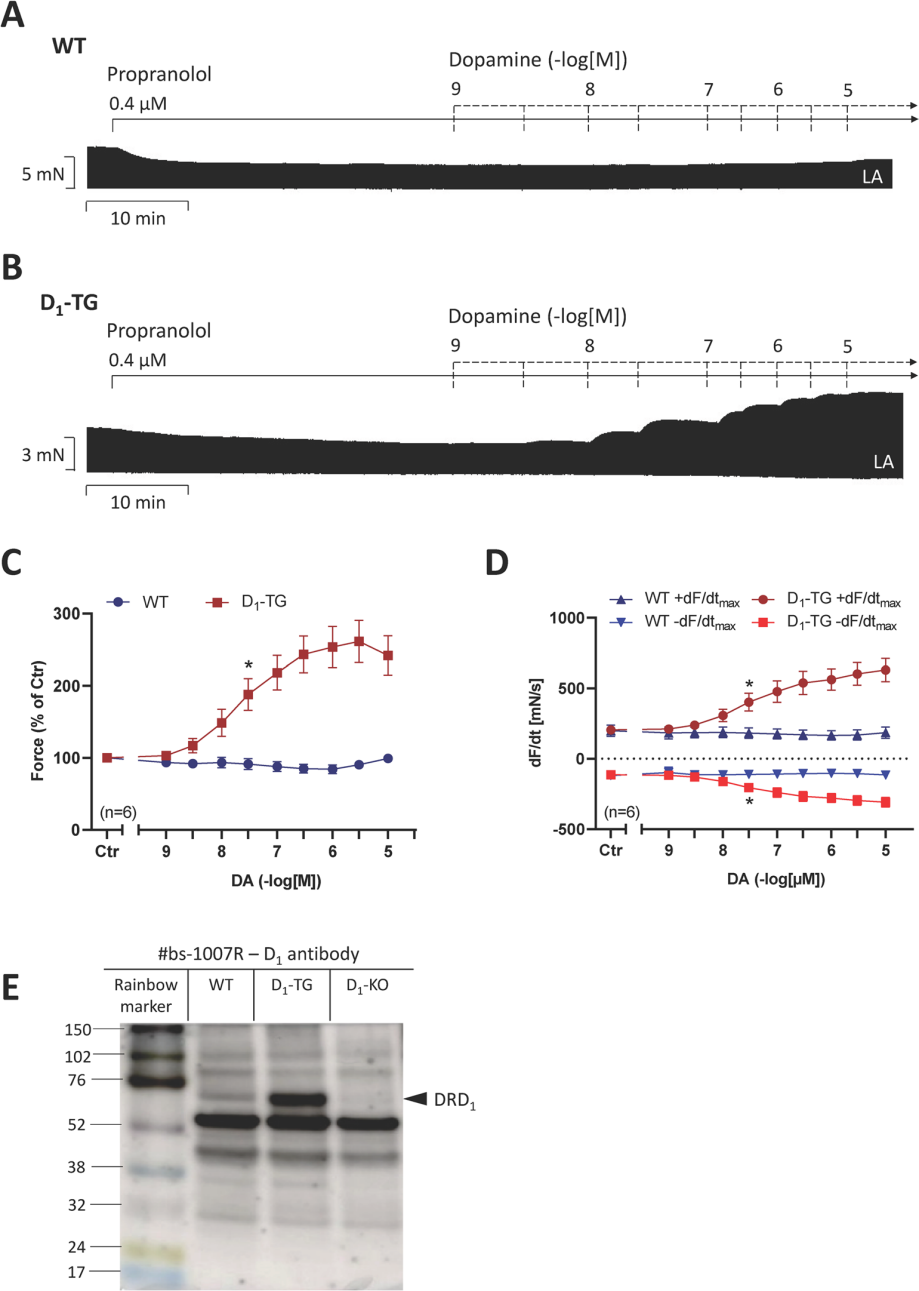
Original recordings in mouse left atrial preparations from transgenic mice with a cardiac overexpression of human D_1_-dopamine receptors (D_1_-TG, **B**) or wild-type mice (WT, **A**). It becomes apparent that dopamine induced a time- and concentration-dependent positive inotropic effect. Summarized concentration–response curves for the effect of dopamine on force of contraction in % of pre-drug value (control: Ctr: **C**), rate of tension development (**D** + dF/dt_max_), and rate of tension relaxation (**D**: − dF/dt_max_). Ordinates in panels **A** and **B**: Force of contraction in mN. Rate of contraction and rate of relaxation in panel **D** in mN/s. Horizontal bars in panels **A** and **B** indicate time bars in min. Abscissae in panels **A**, **B**, **C**, and **D** indicate concentrations of dopamine in negative decadic molar concentrations. Statistical significance was estimated using the analysis of variance followed by Bonferroni’s *t*-test. First significant differences versus control (Ctr; pre-drug value) are indicated by asterisks. Numbers in brackets indicate number of experiments. A typical Western blot is seen in panel **E**. The Western blot depicts the D_1_-dopamine receptor with an arrow (DRD_1_). Relevant molecular weight markers (coloured rainbow markers) are indicated with horizontal lines arrows and are given in kDa. Next lane is sample from WT heart, then sample from D_1_-TG heart and last lane from heart of D_1_-KO mouse (genetic deletion of D_1_-dopamine receptor). The primary antibody was #bs-1007R against the D_1_-dopamine receptor

In the presence of 0.4 µM propranolol, dopamine concentration- and time-dependently increased force of contraction in left atrial samples as shown in Fig. 6B. In left atrial preparations of WT, dopamine did not significantly increase force of contraction (Fig. 6A). This is in line with a previous publication from our group (Rayo Abella et al. 2024), but here we used a different set of animals to corroborate our findings. Moreover, these data indicate that 0.4 µM propranolol is high enough to attenuate any contractile effects of dopamine on β-adrenoceptors. Data in left atrial preparations on force of contraction and the rate of tension development or the rate of relaxation are plotted in Fig. 6C and D, respectively.

**Fig. 7:**
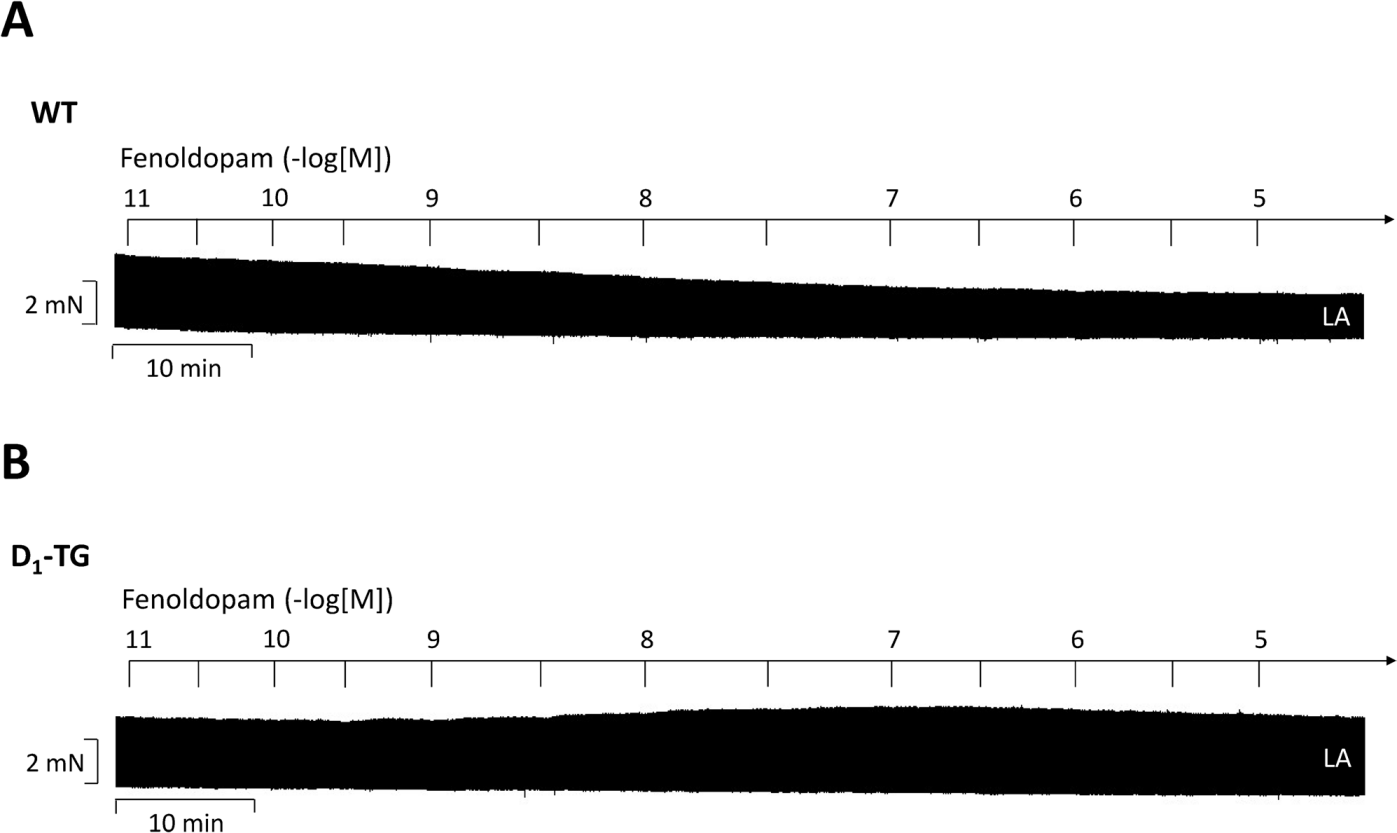
Original recording in mouse left atrial preparations from D_1_-TG (**B**) or wild-type mice (WT, **A**). It becomes apparent that fenoldopam induced a time- and concentration-dependent positive inotropic effect. Ordinates in Fig. 7: Force of contraction in mN. Horizontal bars in panels **A** and **B** indicate time bars in min. Abscissae in panels **A** and **B** indicate concentrations of dopamine in negative decadic molar concentrations

As expected, also fenoldopam increased force of contraction in D_1_-TG but not in WT suggesting to us that fenoldopam acted via stimulation of D_1_-dopamine receptors in the left atrium of D_1_-TG but not WT (original recordings: Fig. 7). These data should be compared to Fig. 5. Figure 7 indicates that fenoldopam alone is in principle an agonist at human D_1_-dopamine receptors when they are overexpressed but does not act in the absence of the D_1_-dopamine receptor in the mouse left atrium.

### Western blot

Similar to previous work, but with different samples, we could detect the human D_1_-receptor in the heart from D_1_-TG. This signal was absent in hearts from D_1_-KO mice and was weaker in sample from a WT mouse heart (Fig. 6E).

## Discussion

### Main new findings

The main new finding of the present paper is our evidence for a direct D_1_-dopamine receptor–mediated positive inotropic effect of dopamine in the human atrium.

Dopamine can increase force of contraction in the human heart. However, the first investigators using selective adrenoceptor antagonists agreed that in the human isolated atrium dopamine activated β_1_- and β_2_-adrenoceptors but not dopamine receptors (Deighton et al. 1992; Brown et al. 1985).

Our present data are based on the inclusion of propranolol in all contraction experiments in order to exclude any action of dopamine on β-adrenergic receptors. Our assumption that we measured effects of dopamine via D_1_-dopamine receptors is based on the selectivity of SCH 23390 to block D_1_-dopamine receptors. As a kind of biological control that our data are valid, we studied the very recently published D_1_-TG (Rayo Abella et al. 2024). In these mice, we expressed the human D_1_-dopamine receptor. Thence, we can rule out any effects of human D_5_-dopamine receptors in D_1_-TG. In contrast, we would argue that under these conditions we can really study dopamine receptor function.

Moreover, others have shown via single-cell polymerase chain reaction in ventricular human cardiomyocytes an expression of D_1_-dopamine receptors which was elevated in failing human hearts (Yamaguchi et al. 2020). However, they did not report contractile studies on these human cardiomyocytes.

There is little evidence in the literature that dopamine can increase force of contraction in the human heart via dopamine receptors. Nevertheless, at least one paper reported that dopamine concentration dependently increased force of contraction in human ventricular muscle strips. This concentration response curve was shifted to the right by haloperidol which can antagonize also D_1_-dopamine receptors (Brown et al. 1985). However, as far as we know, this paper is the first to present evidence for a positive inotropic effect of dopamine in HAP via dopamine receptors and not β-adrenoceptors.

### Clinical relevance

Regardless whether dopamine acts via D_1_-dopamine or D_5_-dopamine receptor, dopamine has a multitude of clinical indications only some of which we may briefly mention in the context of the heart. Dopamine is used to increase force of contraction and improve perfusion of the renal artery in some critically ill patients. The reason for this is usually explained by a stimulation of β-adrenoceptors in the heart and D_1_-dopamine receptors in the renal artery. Fenoldopam is sometimes used to reduce blood pressure in hypertensive patients and thought to act via vascular arterial D_1_-dopamine receptors leading to vasodilation and thus reduced peripheral resistance and thus reduced blood pressure (Myslivecek 2022). Fenoldopam increased the beating rate and prolonged the duration of the action potential in stem cell–derived human cardiomyocytes, which was blocked by SCH 23390, indicating involvement of D_1_ dopamine receptors (Huang et al. 2022). There are clinical studies in which fenoldopam has increased cardiac contractility (Hackman et al. 1992). Hence, our controlled data with fenoldopam might in part explain these results by making a direct positive inotropic effect of fenoldopam via dopamine receptors likely.

In psychiatry, sometimes D_1_-dopamine receptor antagonists are employed. We refer here to the neuroleptic drugs. Some tricyclic antidepressant drugs also block D_1_-dopamine receptors (Garoffolo and Pesce 2021). Hence, when such drugs are given to psychiatric patients, one is also blocking cardiac D_1_-dopamine receptors. Moreover, Morbus Parkinson can be treated by supposedly selective D_2_-dopamine receptor agonists. One has, however, to remember that many D_2_-dopamine receptor agonists are also D_1_-dopamine agonists (Kvernmo et al. 2008; Elayan et al. 1992; Felsing et al. 2019) that may thus act on the heart. In membrane preparations from human hearts, dopamine stimulated the activity of adenylyl cyclases (Amenta et al. 1993, Kaumann et al. 1989). This we have indirectly shown in the present study by measuring an increase in phospholamban phosphorylation. D_1_-dopamine receptor antagonistic effects in the heart may be problematic if D_1_-dopamine receptors were relevant to sustain force of contraction in heart failure patients. At least our work should induce subsequent clinical studies to test our predictions and potentially improve patient care.

## Limitations of the study

In human atrial preparations, we cannot measure the function of the sinus node or the atrioventricular node. Another drawback of our studies lies in the fact that we only studied atrial tissue. It is well known that the receptor expression of the human atrium is different from human ventricular preparations. Hence, the role of D_1_-dopamine receptors in contractile regulation in human ventricular myocardium is currently unknown. However, currently, we have no access to human ventricular preparations and others may try to fill this gap in our knowledge. Moreover, it needs to be elucidated what the general regulatory relevance of the D_1_-dopamine receptors may be in the regions of the human heart in health and disease.

In human atrial preparations, not only D_1_-dopamine receptor but also D_5_-dopamine receptors are present. Only D_1_-dopamine receptors are expressed and are functional in D_1_-TG. Thus, our mouse model does not fully reflect the clinical expression pattern of dopamine receptors in the human heart especially the human atrium. Our data in D_1_-TG are convincing evidence for a possible role of human D_1_-dopamine receptors in the mammalian heart. However, they do not prove that the effects we noticed in the atrium of human heart are D_1_-dopamine receptor mediated. As mentioned in the Introduction, the problem is that D_5_-dopamine receptors are also present in the human heart and there are currently no agonists or antagonists that are specific for D_1_-dopamine receptors (Neumann et al. 2023). D_1_-dopamine receptors and D_5_-dopamine receptors show very high sequence homology although they are coded by different genes (Neumann et al. 2023). It warrants further research effort why such similar receptors are used in nature and are expressed in the same organ.

In any case, one has to wait until others synthesize selective D_1_-dopamine receptor antagonists before progress in this regard can be achieved in the human heart. Our in situ hybridization localized the mRNA for D_1_-dopamine receptor to human atrial cardiomyocytes (Rayo Abella et al. 2024). However, that does not prove but only makes it possible that the D_1_-dopamine receptor as protein is expressed in human atrial cardiomyocytes. These results are only confirmatory: others previously detected D_1_ dopamine receptors in human heart albeit ventricular tissue (Yamaguchi et al. 2020), while we here studied atrial tissue. We are not aware of any published contractile data on an anti-β-adrenergic effect of odapipam, raclopride, and haloperidol in human atrium or mouse atrium. However, this is a possible limitation of our study. This concern underscores the need to study also other more novel putatively D_1_-dopamine receptor selective antagonists for a such off target effects, before they can be used in future contraction experiments in the isolated human atrium to corroborate our present findings.

In summary, we detect a positive inotropic effect of dopamine via D_1_-dopamine receptors in the human atrium that apparently has been overlooked hitherto. This effect of dopamine might be of clinical relevance to explain atrial fibrillation induced by this pathway.

## Supplementary Information

Below is the link to the electronic supplementary material.

Supplementary file1 (**Data 1: **Uncropped Western blot for detection of murine D_1_-dopamine receptor expression in atrial preparations from WT (lane 2), D_1_-TG (lane 3), D_1_-KO mice (lane 4 and 6) and humans (lane 5). As molecular weight marker we used a coloured rainbow marker (lane 1) PDF 196 KB)

## Data Availability

No datasets were generated or analysed during the current study.
