# Supplementary figures and images for: Contractile effects of stimulation of D1-dopamine receptors in the isolated human atrium
Source: Naunyn Schmiedebergs Arch Pharmacol. 2024 Aug 5;398(2):1497–508. doi: 10.1007/s00210-024-03340-z (PMC11825631; doi:10.1007/s00210-024-03340-z)

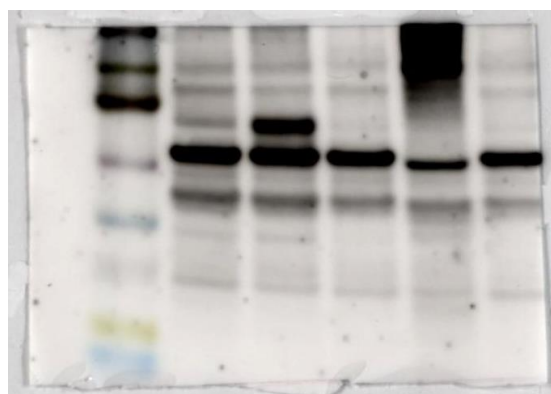

Supplement: Supplementary file 1 — Supplementary file1 (Data 1: Uncropped Western blot for detection of murine D1-dopamine receptor expression in atrial preparations from WT (lane 2), D1-TG (lane 3), D1-KO mice (lane 4 and 6) and humans (lane 5). As molecular weight marker we used a coloured rainbow marker (lane 1) PDF 196 KB) [file 210_2024_3340_MOESM1_ESM.pdf]
